# Immunotolerant Properties of Mesenchymal Stem Cells: Updated Review

**DOI:** 10.1155/2016/1859567

**Published:** 2015-12-29

**Authors:** Whitney Faiella, Rony Atoui

**Affiliations:** Division of Cardiac Surgery, Health Sciences North, Sudbury, ON, Canada

## Abstract

Stem cell transplantation is a potential therapeutic option to regenerate damaged myocardium and restore function after infarct. Current research is focused on the use of allogeneic mesenchymal stem cells (MSCs) due to their unique immunomodulatory characteristics and ability to be harvested from young and healthy donors. Both animal and human studies support the immunoprivileged state of MSCs and even demonstrate improvements in cardiac function after transplantation. This research continues to be a topic of interest, as advances will ultimately enable the clinical use of these universal cells for therapy after a myocardial infarction. Updated *in vitro*, *in vivo*, and clinical trial studies are discussed in detail in the following review.

## 1. Introduction

Heart disease is one of the leading causes of morbidity and mortality worldwide [1]. Specifically, myocardial infarction (MI) remains a significant contributor to cardiovascular related deaths among adults, with 525,000 new and an additional 210,000 recurrent attacks in America each year. It is estimated that approximately 15% of MIs result in death [2]. Following a MI, irreversible ischemic damage may occur to the myocardium if reperfusion is not achieved quickly enough, resulting in reduced ventricular function [3, 4]. Current medical, percutaneous coronary intervention (PCI) and surgical strategies exist to treat coronary artery disease; however, these strategies fail to replace necrotic or scarred myocardium. Stem cell transplantation has emerged as a potential therapeutic option to regenerate damaged myocardium and restore function after infarct [5].

Mesenchymal stem cells (MSCs) are found in bone marrow, adipose tissue, umbilical cord blood, and the placenta of humans [6]. These multipotent cells can differentiate into several lineages including osteocytes, chondrocytes, adipocytes, myocytes, and marrow stroma, which makes them desirable to treat a wide range of chronic and inflammatory diseases [7, 8]. Research into the mechanism of action revealed that MSCs secrete soluble factors such as cytokines and growth factors (prostaglandin, interleukins, tumor necrosis factor stimulated gene, etc.) in a paracrine fashion [9]. More specifically, microvesicles are released from MSCs, carrying mRNA, microRNA, and/or proteins to induce remodelling and a stem cell-like phenotype in injured cells [10, 11]. MSCs can differentiate* in vitro* into myocytes and vascular endothelial cells [12] and can reverse thinning of scarred myocardial regions to improve cardiac function [13].

These cells are particularly desirable due to their unique immunomodulatory characteristics, which allows them to act as a universal or off-shelf reserve and thus eliminates the need for MHC matching prior to treatment [14]. Although autologous stem cell transplantation studies have demonstrated improvements in cardiac function and reductions in infarct size [15], there is a limited capacity for proliferation in cells taken from aged individuals with/without additional comorbidities [16]. The use of cells from young healthy donors for elderly patients in allogeneic transplantations is a promising approach to treat acute MI [17], along with several other diseases.

The immunomodulatory properties of MSCs can be grouped into three categories: being hypoimmunogenic, modulating T cell phenotype, and immunosuppressing the local environment [5]. The following review will discuss updated* in vitro*,* in vivo*, and clinical trial studies with respect to the use of MSCs to improve cardiac function after MI.

## 2. *In Vitro* Evidence

Numerous studies support the immunomodulatory characteristics of MSCs. These cells are known to possess decreased expression of surface molecules including low levels of MHC class I and costimulatory CD40, CD80, and CD86 and no MHC class II molecules [18, 19]. This unique distribution of surface markers allows MSCs to evade detection from certain immune cells and contributes to their hypoimmunogenicity. MSCs are also capable of immunosuppressing the local environment and this can be attributed to their effect on cytokine secretion profiles [20, 21]. Specifically, in coculture with immune cells, MSCs had an indirect effect on T cell maturation and proliferation by upregulating the secretion of suppressive cytokines (i.e., IL-4 and IL-10) to decrease the secretion of proinflammatory cytokines (TNF-*α* and IFN-*γ*) from dendritic cells, T helper cells, and macrophages [22, 23]. Equally important is their ability to induce regulatory T cells, which ultimately inhibits the proliferation and function of T cells, B cells, and natural killer cells [6]. Several soluble mediators including transforming growth factor B1, prostaglandin E2 (PGE2), human leukocyte antigen G5, Haem oxygenase I, nitric oxide, IL-6, and indoleamine 2,3-dioxygenase (IDO) are key to this process [24].

Specifically, PGE2 and IDO are believed to play an important role in the immunomodulation process. Both PGE2 and IDO are involved in the suppression of NK cell proliferation and cytotoxicity, potentially in a synergistic relationship [25]. Aggarwal and Pittenger demonstrated the necessity of PGE2 when the presence of PGE2 inhibitors mitigated the immunomodulatory effects of MSCs [22]. A closer look into the relevant chemical pathways was needed to ascertain the mechanisms involved. It was found that PGE2 induced the secretion of key chemotactic chemokines CCL12 and CCL5. These chemokines are associated with an increased migration of leukocytes to MSCs [26], which is important for stem cells to effectively inhibit their activity [27].

Despite the evidence described above, it has been hypothesized that upon cellular differentiation MSCs may lose their immunoprivileged state; thus, investigations into the properties of undifferentiated versus differentiated MSCs were warranted. The induction of myogenic differentiation with 5-Azacytidine treatment resulted in a >30% increase in MSCs expressing MHC-Ia, a 3–6% increase in cells expressing MHC-II and CD86, and a 30% reduction in cells expressing immunosuppressive MHC-Ib [16, 28]. This change in surface markers was accompanied by a corresponding increase in leukocyte proliferation and activation of CD3+, CD4+, and CD8*α*+ cells in coculture [16]. These results suggest the initiation of an immune reaction upon MSC differentiation. Subsequent* in vivo* studies, which are discussed in detail below, demonstrated rejection of differentiated MSCs by the host [28]. This raises the concern that undifferentiated MSCs may become immunogenic as they differentiate after transplantation, resulting in a loss of therapeutic benefits [6].

Researchers have explored mechanisms contributing to this immune switch and PGE2 has been found to play a role. MSC differentiation resulted in decreased levels of PGE2, chemotactic chemokines, and a conversion to an immunogenic state. The role of PGE2 in this pathway was confirmed when the addition of this factor to differentiated MSC resulted in an increase of chemokines to normal levels and a regain of hypoimmunogenicity [26].

More recent* in vitro* studies have compared the immunosuppressive effects of MSCs from different sources, including bone marrow, adipose tissue, and umbilical cord matrix. MSCs from adipose tissue were shown to exhibit a greater inhibitory effect on B cell function compared to MSCs from bone marrow [29]. Likewise, Ribeiro et al. demonstrated that MSCs from all three sources were all capable of suppressing T cell and NK cell activation but those derived from adipose tissue yielded a stronger effect. Those derived from umbilical cord matrix exhibited no effect on B cell function [30]. More studies need to be completed to ascertain the clinical significance of such findings.

Discovery of the regenerative potential of the heart led to a growing interest in the use of cardiac stem cells for treatment after MI. In 2003, cells isolated from cardiac tissue were shown to differentiate into myocyte, smooth muscle, and endothelial cell lineages [31, 32]. Recent studies have compared the use of stem cells derived from bone marrow versus cardiac tissue for treatment after MI.* In vitro*, bone marrow derived cells showed greater ability to produce adipocytes and osteocytes while cardiac derived cells were more efficient at expressing cardiovascular specific markers [33]. Similarly, stromal cells derived from pericardial adipose tissue exhibited more efficient myogenic differentiation compared to cells derived from inguinal adipose tissue [34]. Perea-Gil et al. characterized a population of mesenchymal-like progenitor cells from cardiac adipose tissue. These cells successfully inhibited T cell alloproliferation in a dose-dependent manner, thus suggesting a new possible source for universal mesenchymal-like cells [35]. Altogether, these results offer new insight that may help to optimize benefits attained from MSC treatment; more insight into the immunomodulatory characteristics of cardiac MSCs is still needed.

## 3. *In Vivo* Evidence

The immunomodulatory properties of MSCs have been well studied and successfully demonstrated* in vitro*; however, there have been mixed findings* in vivo*. Early on, the immunosuppressive effects of haploidentical MSCs were demonstrated* in vivo* when a significant clinical response was observed in treatment-resistant graft-versus-host disease [36]. For the treatment of MI, preservation of left ventricular ejection fraction (LVEF) was found when pigs were directly injected with allogeneic MSCs after MI. No rejection was noted 30 or 60 days after MSC injection [37]. Likewise, an improvement in LVEF from 25.3% to 41.9% within an 8-week period was seen in pigs administered allogeneic MSCs using a percutaneous-injection catheter [38]. In a larger animal model, sheep infused with MSCs after an acute anterior MI had decreased infarct size and an improved LVEF of 52.8% compared to 40.7% in the control group after 8 weeks [39]. These are a few good examples demonstrating the use of allogeneic MSCs as universal donor cells.

There has also been success in xenogeneic models. Bone marrow derived MSCs from mice were successfully engrafted into rats without the use of immunosuppression [40]. Following this study, mouse MSCs injected into an acutely infarcted rat myocardium were tolerated and resulted in improved left ventricular function at 4 weeks [41]. In 2008, human MSCs implanted into rat myocardium were also tolerated and led to improved left ventricular function after 8 weeks [42], thus supporting the feasibility of these universal cells for therapeutic purposes.

On the contrary, there is little* in vivo* evidence supporting the long-term benefit of cellular transplantation. Dai et al. examined the effect of injected allogeneic MSCs in rats after MI on a short- and long-term basis. An improvement in LVEF was noted at 4 weeks but was lost by 6 months. Muscle specific markers, indicative of cellular differentiation, were not fully expressed at 4 weeks but were observed by 6 months [43]. In this study, the improvement in LV function observed before MSCs had differentiated was attributed to an early paracrine effect from transplanted cells. However, other authors dedicate the lack of evidence supporting long-term efficacy and safety of allogeneic MSC transplantation to an immune switch that occurs with cellular differentiation [44].

The retention of immunomodulatory properties upon MSC differentiation is a recent topic of interest. As described above,* in vitro* studies revealed an upregulation of immunologic surface receptors resulting in immune cell infiltration with differentiation.* In vivo*, Huang et al. found that the implantation of allogeneic MSCs into infarcted rat myocardium resulted in improved ventricular function for 3 months. After 5 weeks, anti-donor IgG alloantibodies had formed and reacted with differentiated but not undifferentiated MSCs. At this time, the engrafted MSCs decreased in number and were not detectable in recipient hearts. A decline from the initial improvement in cardiac function was observed after 5 months. Huang et al. characterized this effect using a biphasic model to describe the shift from a hypoimmunogenic to immunogenic state as cellular differentiation proceeded, resulting in a delayed immune rejection/response [16].

Similarly, Xia and Cao found that intramyocardially injected differentiated MSCs were rejected by recipients and resulted in immune cell infiltration in comparison to undifferentiated MSCs. The initial observed improvement in cardiac function was no longer present after 1 month [28]. In a large animal model, pigs received intracoronary injections of adipose tissue derived MSCs after acute MI. The presence of endothelial, smooth muscle and cardiomyocyte markers confirmed cellular differentiation and an immune response consisting of alloantibodies in CD3+ cells was noted [45]. Methods to reduce this immune response have been investigated. For example, the use of a short course tacrolimus delivery prevented a MSC specific immune response when allogeneic cells were transplanted after MI [46].

Given the results described above, more insight into relevant mechanisms was warranted. It was found that an immune switch upon differentiation was associated with decreased PGE2 levels and thus a loss of key chemokine levels. Allogeneic MSC transplantation was performed using a biodegradable hydrogel designed to slowly release PGE2. This prevented the rejection of MSCs after 5 weeks and was accompanied by an improvement in heart function [26]. Therefore, this factor is necessary for MSCs to retain their immunoprivileged state and the use of a PGE2 secreting hydrogel in these experiments offers a possible clinical solution to maximize benefits obtained from allogeneic MSCs [26, 47].

A number of methods for the delivery of stem cells have been explored, including intravenous, transendocardial, and intracoronary approaches. For cardiac applications, cells are typically administered via the transendocardial or intracoronary routes, with studies demonstrating advantages and disadvantages for each approach. Transendocardial delivery provides a greater degree of accuracy since imaging techniques can help identify the exact location for injection [48]. In some of the studies mentioned above, a loss of cardiac benefit was accompanied by a decline in the number of viable MSCs. Specifically, in the experiment by Huang et al., engrafted MSCs decreased in number to 70% of the original value within a week of transplantation and were no longer detectable after 5 weeks [16]. Typically, a decrease in MSCs after intramyocardial injection can be expected due to mechanical leakage and washout [49]. Some authors hypothesize that the loss of cell retention may be a combination of cell death from immune rejection as well as mechanical washout of viable cells [50]. A third hypothesis focuses on complement system activation upon contact of human MSCs with serum, thus leading to MSC injury via membrane attack complexes.* In vivo*, MSCs transferred into complement deficient mice resulted in decreased MSC injury and death in comparison to wild-type mice. This finding indicates the possible need for complement system inhibitors in order to improve viability of MSCs [51]. Regardless of the mechanism, the low retention yield upon intramyocardial injection may account for some contradictory findings in different studies. With this being said, microencapsulation of MSCs is a potential solution to reduce the loss of MSCs after injection [52].

On the other hand, although it is more difficult to deliver cells to the desired region using an intracoronary approach, this method has been associated with good outcomes [53, 54]. Furthermore, intravenous delivery is associated with low retention as cells tend to become entrapped in lung tissue due to their unique size [55]. Differences in host immune responses to transplanted stem cells have not been noted between the various mentioned delivery methods.

Overall, it is important to note that not all* in vivo* studies have demonstrated a loss of MSC immunoprivilege with differentiation. One theory to explain tolerance to MSCs by the host is the “stealth immune tolerance” hypothesis or the “danger model.” These theories suggest that immune reactions occur in response to molecules released from injured tissues rather than markers on foreign or non-self-tissues. For example, in transplantations, rejection might occur from tissue damage during the procedure rather than MHC mismatch [56, 57]. Further studies are needed to clarify the opposing findings, perhaps investigations into the underlying mechanisms and if needed strategies to prevent such immune responses from occurring.

As mentioned above, the use of bone marrow versus cardiac derived stem cells was evaluated.* In vivo*, cardiac tissue derived cells resulted in improved cardiac performance and increased infarcted wall thickness compared to bone marrow derived cells. Cardiac differentiation and arteriole formation were more efficient in mice injected with cardiac versus bone marrow derived stem cells. Specifically, it was noted that cells from the bone marrow failed to form adequate sarcomere structures [33]. In another study, cells from pericardial adipose tissue demonstrated thickening of ventricular wall, vasculogenesis, and myogenesis, ultimately resulting in improvements in cardiac function compared to cells derived from inguinal region [34]. These results indicate potential for the use of cardiac stem cells; however, more evidence is needed, especially studies focusing on the immunomodulatory properties of these cells.

Researchers have even focused exclusively on the use of extracellular vesicles released from MSCs as a therapy to repair damaged tissues. Vesicles isolated from bone marrow derived MSCs demonstrated neoangiogenesis and improved function in ischemic rat hearts [58]. There is potential for these vesicles to be isolated from cardiac stem cells [59].

Despite the hopeful results, the feasibility of harvesting stem cells from cardiac tissue poses a challenge [60]. It has been demonstrated that stem cells can be isolated from cardiac tissue and expanded* in vitro* without losing their potential for differentiation [61]. Therefore, clinical use of these cells might entail isolation and expansion* in vitro*, followed by transplantation. Since resident cardiac stem cells may have limited regenerative potential in damaged tissues after MI, the use of allogeneic cardiac stem cells remains a hopeful therapeutic option but isolation methods must be first optimized.

## 4. Clinical Trials

Clinical trials, including the LateTIME, the TIME, and the Swiss Myocardial Infarction trials, tested the use of autologous MSCs for the treatment of acute MI [62, 63]. There are only a few documented trials examining the use of allogeneic MSCs for the same purpose. In 2007, phase I trial sponsored by Osiris Inc. demonstrated improved ventricular function after 6 months in patients who received intravenous infusions of allogeneic MSCs [64]. The same company developed Prochymal, human mesenchymal stem cells for IV infusion, which received conditional approval in Canada and New Zealand in 2012 for the treatment of graft-versus-host disease [65, 66]. In 2009, phase I trial evaluated the safety and efficacy of intravenously delivered bone marrow derived allogeneic MSCs after MI. There were similar occurrences of adverse events, with noted improvements in global symptom score and ejection fraction in the treated versus control groups after 3 and 6 months. Interestingly enough, MSCs were infused into patients at three different doses; however, dose-dependent effects in most parameters, including LVEF, were not observed [67]. Nevertheless, these results demonstrate promising findings and reiterate the need to define the safety and efficacy of treatment over a longer period of time.

The POSEIDON randomized trial compared the use of allogeneic versus autologous bone marrow derived MSCs, as a phase I/II trial, in patients with ischemic cardiomyopathy over a 1-year period. There was a lower 1-year incidence of serious adverse events, including immune responses, in the allogeneic (33.3%) versus autologous (53.3%) cell treated groups. After 1 year, improvements in patient function and ventricular remodeling were also noted (i.e., reduced infarct size and improved sphericity index) [68]. An additional imaging study was performed to determine if a relationship existed between the site of MSC injection and sites of improvement in myocardial scar and ejection fraction. A reduction in infarct scar size occurred at both injected and noninjected sites; however, more of an increase in LVEF was noted in injected scar segments [69]. These results are a good start for future investigations to optimize the delivery of MSCs. Furthermore, a related POSEIDON-DCM trial, currently in phase I/II, was designed to examine the safety and efficacy of autologous versus allogeneic bone marrow derived MSC injections in patients suffering from nonischemic cardiomyopathy [70]. If successful, this study can expand the pool of patients eligible for stem cell therapy.

In a more recent trial, allogeneic MSCs were injected into the myocardium during left ventricular assist device (LVAD) implantation. After 90 days, successful temporary LVAD weaning occurred in 50% of patients treated with MSCs in comparison to only 20% in the control group. However, 12 months after injection, these values were 30% and 40% in the treated and control groups, respectively. LVEF after 90 days was 24% versus 22.5% in the treated and control groups, respectively. Authors concluded that MSC therapy appeared to be safe, with potential benefit for cardiac function; however, more studies are needed to determine optimal dosages [71]. Again, the lack of benefit in the treated group after 12 months reiterates the need for further investigations focusing on long-term efficacy of this therapy.

In September 2014, the safety results from the Allogeneic Heart Stem Cells to Achieve Myocardial Regeneration (ALLSTAR) trial were revealed. In this phase I trial, allogeneic cardiosphere-derived cells were administered to patients after MI via intracoronary infusion. In terms of immune reactions, donor specific antibody levels remained low with no detected cellular immune responses after infusion [72]. This study has advanced to phase II and shows promising results given the evidence supporting the regenerative potential of cardiac derived stem cells and their immunotolerant properties.

Further clinical trials evaluating the safety and efficacy of allogeneic mesenchymal precursor cells for myocardial infarction are currently in progress. The AMICI trial is in phase II and will administer bone marrow derived cells percutaneously in patients with an anterior MI [73]. A second trial is designed to evaluate the safety and feasibility of transendocardial delivery of cells using mapping and injection catheters in patients with acute MI [74]. These trials will hopefully offer valuable results to potentiate the clinical use of stem cells for cardiovascular disease.

## 5. Conclusion

MSCs continue to be investigated as a future therapeutic option to regenerate damaged myocardium and restore function after infarct. Their unique immunomodulatory characteristics and the potential for allogeneic cells to be harvested from young and healthy donors make these cells desirable for cellular transplantation. Numerous studies support the immunomodulatory characteristics of MSCs* in vitro* but there have been mixed findings* in vivo*. More specifically, there is controversy surrounding the possibility that MSCs may lose their immunoprivileged state upon differentiation, thus triggering an immune response and rejection by the host after implantation. Furthermore, clinical trials are currently in phases I and II and have demonstrated promising results. Overall, this research offers a possible new approach to manage myocardial infarction that may be used concurrently with medical, percutaneous coronary intervention and surgical strategies. Future research should focus on optimizing parameters including source of cells, timing of injections after MI, and delivery methods, as well as establishing long-term efficacy.
